# No Laughing Matter: Presence, Consumption Trends, Drug Awareness, and Perceptions of “Hippy Crack” (Nitrous Oxide) among Young Adults in England

**DOI:** 10.3389/fpsyt.2017.00312

**Published:** 2018-01-22

**Authors:** Esther M. Ehirim, Declan P. Naughton, Andrea Petróczi

**Affiliations:** ^1^School of Life Sciences, Pharmacy and Chemistry, Kingston University, Kingston upon Thames, United Kingdom

**Keywords:** hippy crack, nitrous oxide, laughing gas, prevalence, legal high, novel psychoactive substances, harm-reduction

## Abstract

In clinical settings, nitrous oxide gas is a safe anesthetic used during childbirth, in dentistry, and to relieve anxiety in emergencies. Colloquially known as “hippy crack”’ or “laughing gas,” it is increasingly taken recreationally for its euphoric and relaxing effects and hallucinogenic properties. Using a self-reported survey, we gathered quantitative and qualitative information on users and non-users of hippy crack among a young population regarding: consumption patterns, knowledge, risk awareness and intentions toward future abuse. Quantitative responses from a total of 140 participants were analyzed for frequencies and relationships, whereas qualitative data were evaluated *via* identifying the reoccurring themes. Overall, 77.1% (*n* = 108) had heard of hippy crack and 27.9% (*n* = 39) admitted to past-year use. Prior users mostly indicated intended future use, had an average low number of past-year uses but some with > 20 occasions, had a varied number of inhalations per occasion (often 1–10) with an effect lasting up to 5 min, and a majority preferred social rather than lone use. For non-users, 79.2% said they would take hippy crack with the vast majority (94%) preferring a social setting. The results show a concerning gap between available evidence and awareness of side effects. Despite serious reported side effects, including psychosis and myeloneuropathy—especially on the young developing brain—only a minority (29.3%) was aware of any side effects. In contrast, in a hypothetical scenario depicting a first social encounter with hippy crack, the qualitative responses were in contrast to qualitative outcomes revealing that participants would try (*n* = 30)/not try (*n* = 25) it, would feel under pressure to try it (*n* = 6) with only 11 opting to exit the situation. In summary, this first report of trends and perceptions of the use of hippy crack among young adults in the England highlights a lack of concern with side effects, coupled to a willingness to partake. Because typical users are young with risks to the still developing brain, education about the nitrous oxide abuse is warranted to prevent impaired brain development. Further studies to investigate the possible effects of nitrous oxide on the developing brain in young adults would advance meaningful prevention.

## Introduction

Hippy crack or laughing gas (nitrous oxide, N_2_O) is used clinically as a safe anesthetic, allowing pain relief during childbirth, in dentistry, and to relieve anxiety in emergencies (1, 2). Nitrous oxide in gaseous form, once inhaled, dissolves in the bloodstream, reaching the brain within seconds. Being more water soluble than oxygen, it will diffuse more rapidly across the alveolar basement membrane, resulting in rapid entry into the bloodstream causing dilution of the volume of the gaseous contents (e.g., oxygen) of the alveolus (3, 4). In consequence, diminished oxygen levels (hypoxia) *via* the decrease in alveolar oxygen tension can subsequently decrease oxygen delivery to the brain. A laboratory simulation confirmed that nitrous oxide displaces oxygen in a closed space leading to asphyxia (5), which occurs when inadequate amounts of oxygen are supplied to the tissues and organs.

Furthermore, studies involving the use of nitrous oxide show it causes vitamin B12 (cobalamin) deficiency (6). Nitrous oxide irreversibly binds to the cobalt ion within vitamin B12, causing the inactivation (2). Vitamin B12 deficiency has been correlated with demyelination of nerve cells (7). In addition to nitrous oxide-mediated consequences of hypoxia and vitamin B12 deficiency, hippy crack leads to an increase in homocysteine, a *N*-methyl-d-aspartame agonist associated with oxidative stress and mitochondrial disruption *via* intracellular calcium release (2, 8). Abuse of hippy crack has been reported to cause fatalities (5, 9–12), and—in association with low or low-normal levels of vitamin B12—to psychiatric effects such as psychosis, peripheral neuropathy and other medical effects relating to blood flow (13–18). Extreme case reports have involved subacute combined spinal cord degeneration and ataxia following nitrous oxide abuse (19–21).

Brain development from age 18 to 25 years specifically involves the rewiring-process within the prefrontal-cortex, which develops last (22). The prefrontal-cortex role involves, obtaining information from all of the senses and coordinating thoughts and actions, giving an individual the ability to utilize good judgment when presented with difficult life situations *via* abstract thought (22). The rewiring-process involves dendritic pruning, cutting and eradicating unused synapses (23) and myelination (22), involving the accumulation of the myelin sheath, the myelin-forming cells (Schwann cells), increasing the speed, and propagation of impulse conduction across the brain (23). Vitamin B12 deficiency—a result of repeated nitrous oxide intake—will likely impair these processes through multiple effects especially defective myelination.

However, hippy crack remains popular among young people and—with ease of accessibility—is increasingly being taken recreationally (2). According to the Global Drug Survey (24), 91% of users used once or less per month and 64% of users take of up to five balloons per session (25). The following year, 4% of users reported symptoms consistent with nerve damage (24). Serious short-term reversible effects have been reported with case reports describing use of up to 100 cartridges per day over several months (26). In the United Kingdom, legislation has been introduced to ban psychoactive substances and to prevent supply of these substances for human consumption. Nitrous oxide was specifically included in the “Guidance to Retailers” accompanying the Psychoactive Substances Act (27) along with other legal highs/novel psychoactive substances. A recent report reveals nitrous oxide is the seventh most popular drug worldwide and there is increasing use of nitrous oxide in the UK with a 38.6% lifetime prevalence by 2014 (24, 28). However, no in depth study has looked into the effects on the developing brain from 18 to 25, an age group possibly more likely to recreationally consume this drug. The overuse of hippy crack may slow down if not reverse the progress of the developing brain *via* effects on vitamin B 12. What remains unknown is how much is too much and how this level compares to the typical recurrent use less than monthly and up to five balloons per occasion.

Thus, it is timely to explore how popular hippy crack is among young people (18–25 years), as well as to decipher the consumption pattern of the drug taken recreationally, in addition to measuring people’s actual knowledge of the drug and what it does. Using self-reported surveys, we aimed to gather quantitative and qualitative information on users and non-users of hippy crack among a young population regarding: consumption patterns, knowledge, risk awareness, and intentions toward future abuse.

## Materials and Methods

A survey approach was used to explore how aware young people are of the harmful effects of the drug itself. Furthermore, exploring young peoples’ susceptibility to using the drug as to whether it is a case of simple enjoyment or whether recreational use is more popular as a social activity, bringing light to how people are likely to behave in a given situation. In addition, we employed a qualitative approach using a hypothetical scenario depicting a plausible first social setting encounter to investigate attitudes toward potential first use.

### Participants

Data were collected from 140 participants in south west London during January and February 2017. Participants were recruited randomly using a combination of hard copy or electronic copy *via* email (*n* = 130) within Kingston University and online *via* survey monkey (*n* = 10). The content of the surveys was identical (details of the full survey appear in Annex 1). Participants were in the age range of 18–25 years with 94 females, 40 males, and 6 not declaring gender. The study was approved by the delegated approval scheme of the Faculty Research Ethics Committee of the Faculty of Science, Engineering and Computing, Kingston University.

### Measures

The five primary outcome measures include: presence, knowledge of the drug, hippy crack intake, behavior, and risk. A range of secondary outcome measures were also assessed to help determine the consumption pattern of hippy crack for 18–25 year olds. Response options in closed questions ranged from 1 to 10 and 1 to 5 (on various answers capturing willingness, likelihood, agreement and risk perception), with some only offering dichotomous response options (yes and no). In addition, a hypothetical scenario was used to assess participants approach to trying hippy crack to allow participants to mentally put themselves in a situation in which they are presented with hippy crack. The quantitative scenario question was complemented with an open answer option, which prompted participants to elaborate on their scaled quantitative response. The full survey is detailed in Annex 1.

### Scenario-Based First Encounter

A social encounter with encouragement to take hippy crack based on a scenario was used to elicit attitudes toward potential use. A gender-neutral name (Alex) was used to avoid gender bias and allow respondents from both genders to identify with the protagonist. The scenario question also explores perceptions of risk (Annex 1).

### Data Analysis

For quantitative data, descriptive statistics was used to obtain mean, highest and the lowest value and the SD. Chi square test was used to test relationship between categorical responses (e.g., between two groups/variables such as gender and whether they have taken hippy crack). IBM SPSS statistics version 23 was utilized. The open question was analyzed manually using thematic analysis to identify repeated words/phrases (“peer-pressure,” “knowledge,” and “risk”) and potentially emerging new themes between individuals in addition to observing frequency of the reoccurrence of that phrase.

## Results

### Quantitative Survey

The majority of participants were female (67.1%, *n* = 94) with gender not declared for 4.3% (*n* = 6), with ages between 18 and 25 years (mean age of 21 ± 1.70), and 94.6% were students (*n* = 135). The majority (77.1%, *n* = 108) had heard of the drug hippy crack and 27.9% (*n* = 39) had taken hippy crack in the past 12 months. Hippy crack consumption was more popular among males at 39.0% (*n* = 16) compared to females at 24.7% (*n* = 23) but did not reach statistical significance [χ^2^ (2) = 5.31, *p* = 0.07]. Non-users in the past year comprised of 75.3% (*n* = 70) females and 61.0% (*n* = 25) males, with participants over the age of 20 being more likely to take hippy crack. The group aged 18 (*n* = 9) had no report of taking hippy crack. There was no statistically significant association between age and consumption of hippy crack [χ^2^ (7) = 9.68, *p* = 0.208].

### Outcomes: Hippy Crack Users

**Figure 1:**
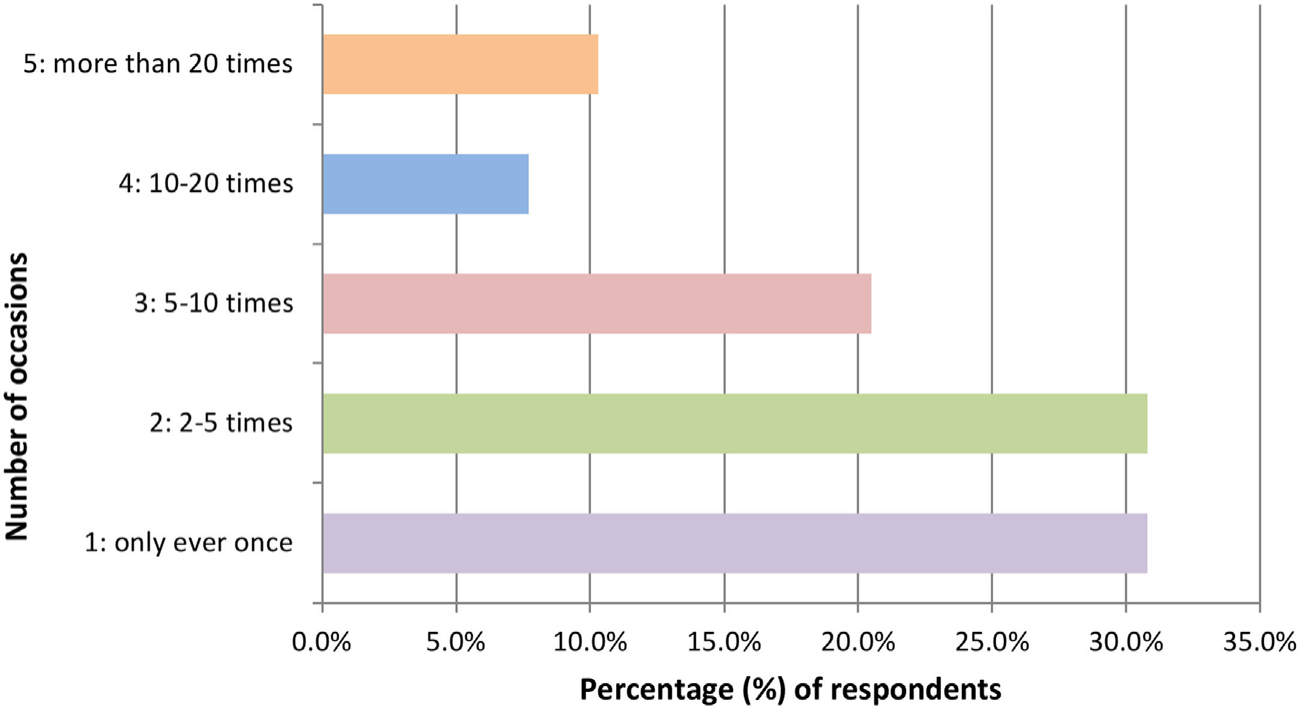
Number of occasions hippy crack was taken in the past year (*n* = 39 responses).

**Figure 2:**
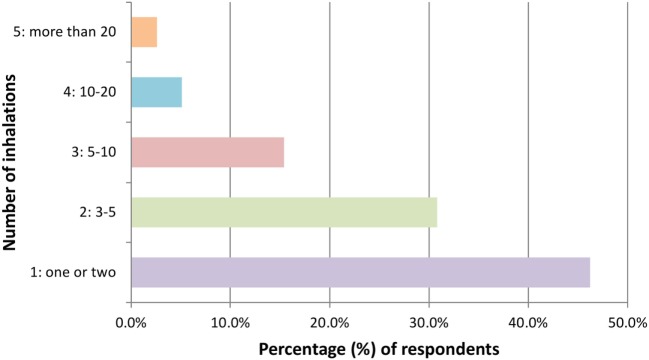
Quantities (inhalations) of hippy crack inhaled in one sitting (*n* = 39 responses).

**Figure 3:**
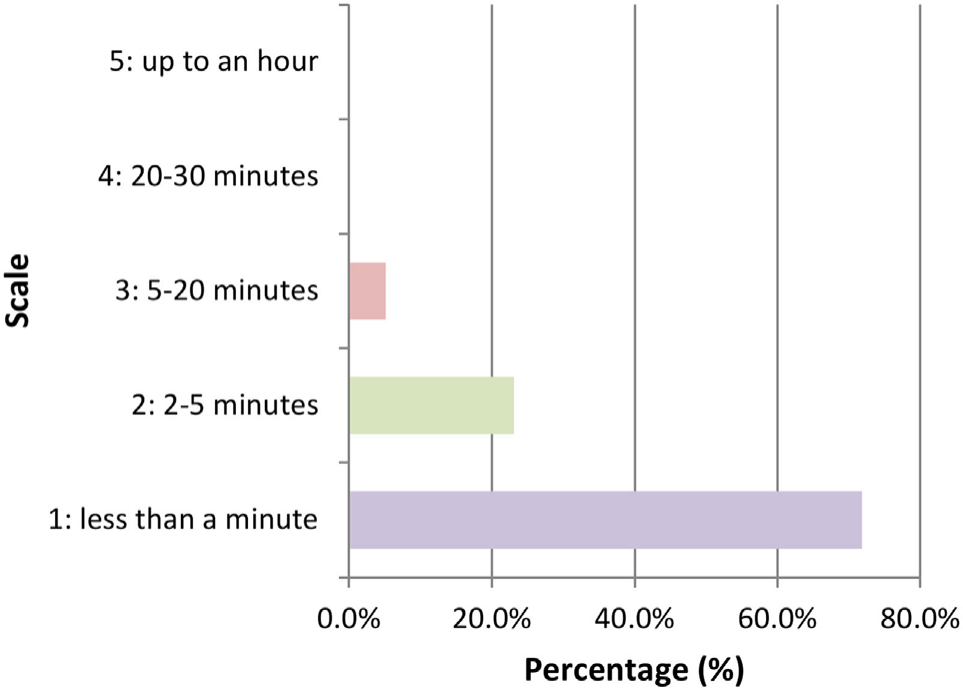
Duration of the effect after inhalation of hippy crack (*n* = 39 responses).

Of the 39 participants reporting previous experience of using hippy crack, the majority (*n* = 27) took it on more than one occasion during the past year. Notably, only 7 users had taken it at or exceeding 10 occasions reinforcing the majority of respondents being light users in line with previous reports (6) (Figure 1). The amount taken on each occasion varied with 46.2% (*n* = 18) partaking once or twice in one sitting but for the majority this extends to ≥3 intakes and even greater than 20 intakes (Figure 2). Furthermore, users are more inclined to take hippy crack among friends, 97.43% (*n* = 38) than on their own. Most users (87.2%, *n* = 34) reported an effect after one or two intakes that lasts for up to 1 min (Figure 3). Most users reported the ease of acquisition of hippy crack (57.15%, *n* = 28). Many past users, indicated that they would take hippy crack again in the next 3 months (scale = 1 absolutely not, 5 definitely, average 3.38, most common = 5). Those over the age of 20 were more likely to reuse hippy crack, with participants aged 21 years being most likely to retake hippy crack (46.7%, *n* = 7). A statistically significant association was found between age and likelihood to retake hippy crack [χ^2^ (24) = 79.442, *p* = 0.001].

### Outcomes: Non-Users of Hippy Crack

**Figure 4:**
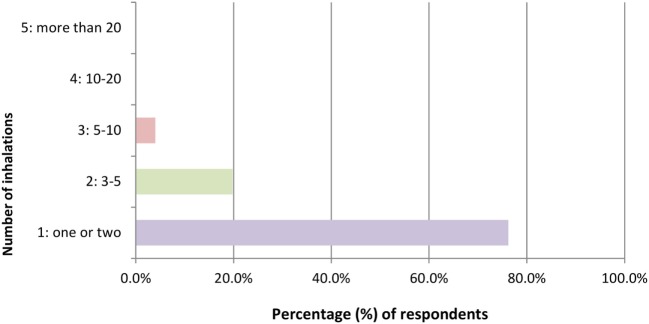
Number of inhalations of hippy crack that would be taken in one sitting if one has never taken hippy crack (*n* = 101 responses).

For non-users (*n* = 101) of hippy crack in the past 12 months, the likelihood of trying hippy crack in the next 3 months was very probable (79.2%, *n* = 80), with the mean scale value being 4.49 ± 1.11 (where 1 means very unlikely and 5 means very likely). Of the non-users, the majority (76.2%, *n* = 77), would have hippy crack one or two times, with a minority (4.0%), indicating using hippy crack 5–10 times with no one considering over 10 times (Figure 4). Many non-users also reported perceived ease of supply with 37.62% (*n* = 38) reporting it as “very easy.” In addition the majority (93.9%, *n* = 92) would be more comfortable taking hippy crack among friends than on their own.

### Harm Awareness and Reduction

**Figure 5:**
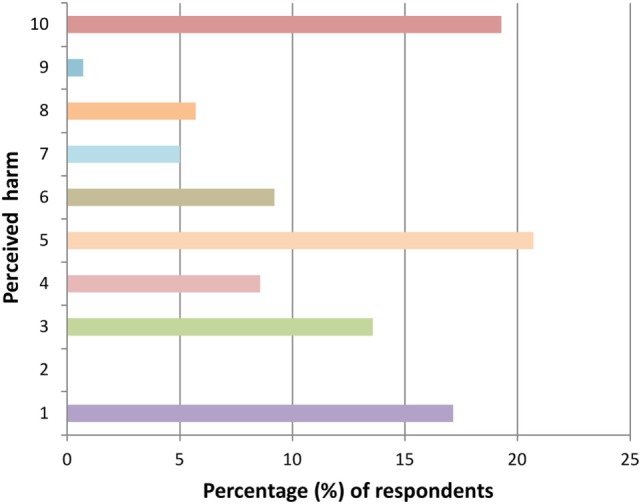
Perceived harm from using hippy crack (10 = extremely harmful) (*n* = 140 responses).

**Figure 6:**
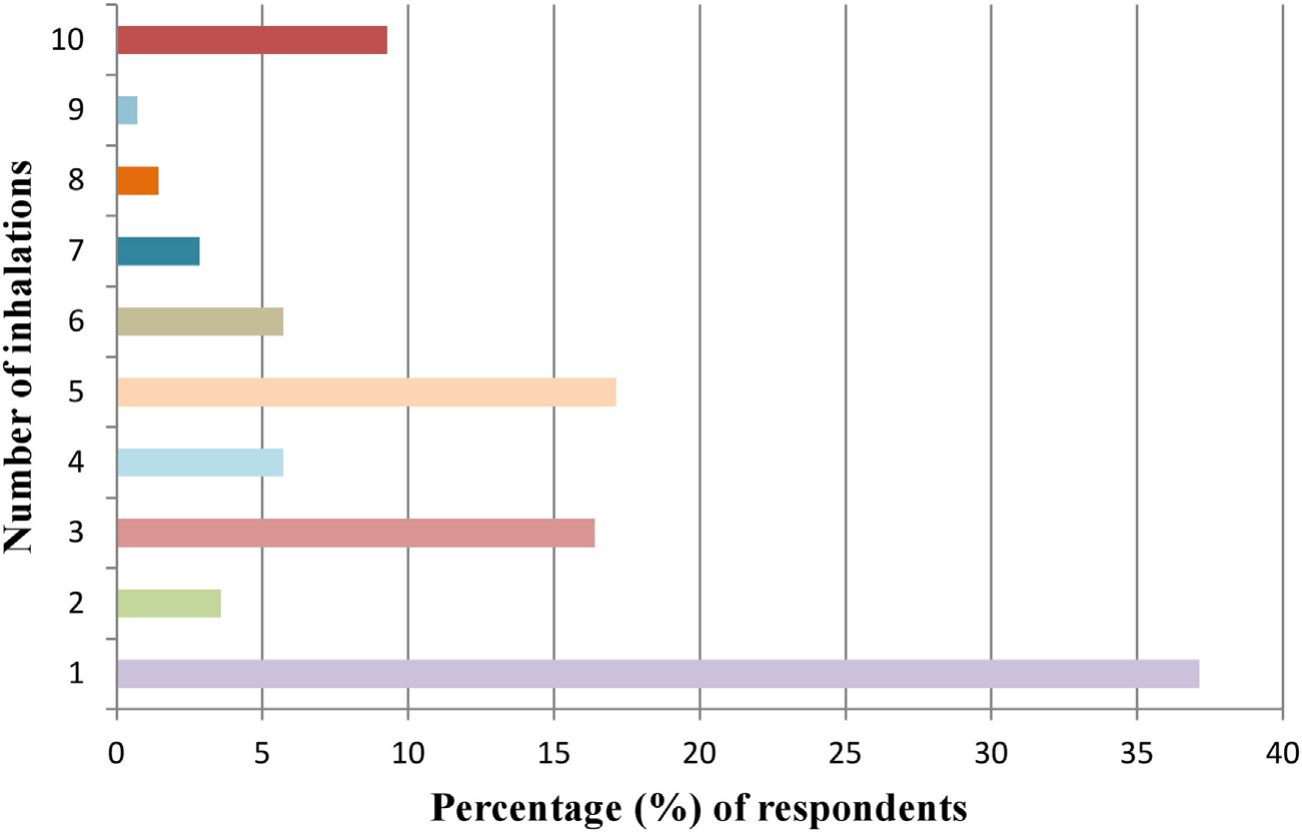
Number of inhalations perceived as enough hippy crack for a single occasion (*n* = 140 responses).

Perceptions of the level of harmful effects of hippy crack varied widely with a full scale response between 1 (not harmful at all) and 10 (extremely harmful) with the mean scale value being 5.31 ± 3.01 (Figure 5). Additionally, the majority (91.6%, *n* = 99) of those who had heard of hippy crack were not aware of any side effects associated with hippy crack use. Views regarding how much hippy crack should be consumed in one setting were diverse with a full range from 1 to 10 (the maximum given) (Figure 6).

A clear message was received regarding the importance of teaching young people about the harmful effects of hippy crack. The majority (60%, *n* = 86), chose the scale value of 10 (extremely important) as to how important it is to educate young people about the effects of hippy crack, with the mean scale value of 8.43 ± 2.47 emphasizing a willingness to learn about the risks.

### Qualitative Scenario Setting

The scenario setting extension to the self-report was designed to encompass wider views based on a plausible first encounter with hippy crack. The approach taken was to elicit attitudes toward the offer of hippy crack, along with awareness of risks of consumption in confined spaces. Themes emerging, from the qualitative responses to the scenario question are shown in Table 1, which is further split into content subgroups and frequency, showing how often content is repeated.

**Table 1:** Summary of qualitative results, behavior of those offered hippy crack, susceptibility to risks, knowledge of hippy crack.

Category (themes)	Content	Frequency
Behavior of those offered hippy crack	Would take hippy crack just to try it	30
	Experience	9
	Feeling of being under (peer pressure)	6
	Intake of hippy crack dependent on level of comfort	4
	Go home/leave	11
	Simply would not take hippy crack	25

Susceptibility to risks involved with hippy crack	Inhale in the car	8
	Sit in the car even though they stated that they would not inhale hippy crack	5

Knowledge of hippy crack	Inhaling outside the car	26
	Just inhale hippy crack *via* balloon in the house	3
	Inform friends of possible dangers involved with inhaling hippy crack	21
	Change their minds due to known information	28

In-depth analysis of the qualitative responses revealed three overarching themes: intention to use hippy crack, knowledge and acting on knowledge, and susceptibility to risks.

### Taking Hippy Crack

Those who have not taken hippy crack and answered no, were actually more likely to have taken hippy crack if offered, the word “*try*” and “*experience*” were most common (“*try it just for experience*”) in responses, which could be due to lack of knowledge, therefore, less skeptical/less cautious and more likely to try hippy crack, even if it’s just due to curiosity (“*go for it man has nothing to lose, it*’*s not the things we have done in life that we think about when we die, it*’*s the things we didn*’*t do that haunts us*.”). A few participants stated if effects were euphoric enough chances of use may increase (“*depends on if the affect was deemed worthy enough*”), moreover, this could lead to a higher consumption pattern.

Some people stated that they would try just the one or at the very least a small amount (“*Try maybe half a balloon just to be sure*”). However, taking even a small amount may lead to them taking more once under the influence, still leaving them at risk, so they are still susceptible to harm. Furthermore, for those willing to take in the car with windows down, it is more likely, they could be persuaded to inhale hippy crack with the car windows up once under the effects of the drug, as judgment may be altered, momentarily, friends may close the windows in a fit of laughter thus leaving them more susceptible to danger.

As the scenario question is set as Alex’s first encounter—some responses were more cautious, as to whether they would take hippy crack as it would be their first try (“*Sounds too much for the first try*”). However, if one was more experienced/taken hippy crack before, is it more likely that they would have taken hippy crack once offered, perhaps due to feeling less hesitant as according to results. There are some participants that stated that they would still go along with them/sit in the car, even though they would be releasing nitrous-oxide into the car (a closed space), consequently they could still be susceptible to harmful effects of hippy crack, moreover them being cautious, still leaves them vulnerable (“*wouldn*’*t take it but go along with them bearing in mind that it could cause side effects*”).

### Knowledge of Hippy Crack

Uncertainty because of lack of information showed to be quite common (“*I think there needs to be more awareness on all drugs at this age, it is very common for young people to take drugs not being aware if the risks involve*d”). People could also be dissuaded due to the little knowledge they’ve obtained from the scenario question (“*never intentionally take drugs, knowing it could have an effect on me*”), suggesting knowledge of hippy crack can indeed have an effect on one’s consumption pattern. Some participants said they would not do it and leave, however, others, instead of just leaving, would also warn their friends of possible dangers involved in taking hippy-cark (“*inform my friends of side effects*”) (Table 1). This suggests that the more people that are informed, the more likely they would share this information with others among their peer group, increasing knowledge and awareness of hippy crack, further suggesting that informing people of risks could result in them and those around them being less susceptible to risks/side-effects, overuse of hippy crack and at the very least making people take hippy crack in a safer way (“*Tell my friends not to roll up the windows*”), possibly even reducing consumption pattern. A number of participants suggested taking hippy crack in a safer way by taking it outside (“*open environment*”), which could lower possible risks leaving them less susceptible to side-effects. Further emphasizing that knowledge of the drug could be beneficial itself as it could alter the way it is taken.

### Susceptibility to Risks Involved with Hippy Crack

Furthermore, some people do not see the importance of the harmful side effects hippy crack may cause, neglecting information/warnings given in the scenario question (“*go for it, there*’*s no strong evidence to suggest its damaging to you moreover, the high only last a few seconds*”). Even though effects of hippy crack were reported to last a few seconds, people may not be aware of hippy crack causing a lack of B12, increasing levels of homocysteine in the blood, which causes more harm, as it can take up to days to clear. Additionally, due lack of knowledge, people may not view hippy crack as something worth being cautious over and may have a relaxed attitude toward the drug (“*yolo, you only live once so why not*”). This lack of awareness of risk could increase consumption pattern leaving them more susceptible to side-effects/risks.

## Discussion

This is the first study to capture a wide range of key characteristics of both users but also potential future users to inform future strategies to educate, avoid harm and prepare young people for the risks involved in the regular abuse of this seemingly harmless legal high. At a time when little is known about the effects of hippy crack on the developing brain, it is very concerning that the majority of participants showed little or no knowledge of serious side effects associated with this drug. Previous studies have largely focused on quantitative data such as prevalence without gaining a fuller appreciation for the behavior and future intentions of young people.

The current study used convenience sampling whereas prior study used a slightly older age range and targeted recruitment (purposive sampling). Thus, the key findings of the current study are complimentary in highlighting, in particular, the desire to partake of hippy crack for both non-users and last-year users and, the lack of awareness of potential side-effects and the desire to be educated on the effects of hippy crack. It is notable, that despite the introduction of legislation by UK Government, this report testifies to the relative ease in which young people assert that they can access hippy crack. A further complication arose in 2017 when—owing to its medicinal properties—a UK judge dismissed a case of festival attendees possessing hippy crack (29).

This study aimed to assess the widespread abuse of hippy crack along with the issues related to its “silent” toxicity profile. Increased understanding of attitudes to—and involvement in—hippy crack abuse among young adults is importance to health issues—both physical and mental—along with fatalities. The lack of awareness of the associated serious side-effects lends perspective to the need for increased education of young people, their guardians and health workers. Key findings of this study included the paucity of understanding of the toxic effects of hippy crack alongside a widespread desire for education for harm-reduction. For example, in severe cases of hippy crack-induced vitamin B12 deficiency, serious symptoms could be reversed with administration of vitamin B12 in a clinical setting (30). With appropriate education and risk awareness, users would be empowered to protect their health—both mental and physical.

Previous research has ascertained the prevalence of use across six nations with the United Kingdom leading for lifetime, last year and past month use (38.6, 20.5, and 7.7%, respectively) (28), which is slightly below the past-year use (20.5 vs. 27.9%) identified in the current study. It identified the main sources of purchase (internet), routes of administration (by mouth *via* balloon), and location of use (house parties). The authors further focused on adverse effects last-year users had experienced along with their health concerns. Furthermore, inhalants use among adolescents and young children is a growing public health concern (31, 32). Evidence from previous research also suggests that inhalants, including nitrous oxide, are often the first category of substances to be abused by adolescents and thus considered a ‘gateway drug’ (33–35); and that the growing popularity of e-cigarettes among adolescents increases the relative risk for using nitrous oxide (36).

This study has limitations in that the results are restricted to a relative small sample size and the restrictions associated with self-report measures. Participants could not give a verbal confirmation when using number scales as answers. The closed questions (selection of yes or no) can be considered to be limited in terms of data, which in turn could have affected the precision of the overall data obtained. However, by using the scenario/open question a reasonable idea as to how people would behave in a given situation, in addition to, helping to understand a little more about how people may view the drug itself, in terms of the harm the drug can cause and also how they consider risks associated with the drug.

## Conclusion

These results provide considerable insights into the level of use, awareness and perceptions of hippy crack in young people in England. The reported use (number of occasions and amounts taken), coupled to the apparent willingness of most respondents to use it in the future, is overshadowed by the clear lack of awareness of the serious side effects it may cause. Holistically this report emphasizes the growing need to educate people on the dangers involved upon consumption of hippy crack especially as it appears that the new Psychoactive Substances Act is failing to curb abuse. A drive toward harm-reduction is necessary through dissipating the widespread belief that this “legal high” is without side effects. Furthermore, empirical studies should be designed to address the effects of hippy crack on the developing brain—based on the reported considerable molecular and structural changes that result from frequent use.

## Ethics Statement

The study was approved by the Faculty Research Ethics Committee of the Faculty of Science, Engineering and Computing, Kingston University under the delegated approval scheme. To ensure complete anonymity, implied consenting process was used. At the start of the data collection, participants were informed about the study, the use of data, the data collection process and they gave consent by voluntarily completing and returning the survey.

## Author Contributions

AP and EE made substantial contributions to conception and interpretation; drafting and critically revising the work for important intellectual content, final approval and agreement to be accountable for all aspect of the work. DN made substantial contributions to drafting and critically revising the work for important intellectual content, final approval, and agreement to be accountable for all aspect of the work.

## Conflict of Interest Statement

The authors declare that the research was conducted in the absence of any commercial or financial relationships that could be construed as a potential conflict of interest.

## Annex 1. Survey Questions

1. Gender ◻ male ◻ female ◻ neither ◻ other ◻
2. Age in years ◻
3. Student: yes or no
4. Have you heard of hippy crack before this questionnaire? yes or no
5. Have you taken hippy crack recreationally in the past 12 months? yes or no
  If you have answered **yes (have taken hippy crack)** answer questions **1–13**
  If you have answered **no (haven’t taken hippy crack)** answer questions **14–17**
  **All**, answer questions from 18–22
  **If you answered YES**
6. If you have taken hippy crack before, would you take hippy crack again within the next 3 months?
  Scale 1–5
  1: absolutely not
  5: definitely
7. If you wanted to, how easy would it to obtain hippy crack?
  Scale 1–5
  1: very easy
  5: very difficult
8. How many times would you say you have taken hippy crack in the past 12 months?
  Scale 1–5
  1: only ever once
  2: 2–5 times
  3: 5–10 times
  4: 10–20 times
  5: more than 20 times
9. In your opinion, would you say you more likely to take hippy crack on your own or among friends?
  ◻ On my own
  ◻ Among friends
10. How much hippy crack would you inhale in one go/sitting?
  Scale 1–5
  1: one or two
  2: 3–5
  3: 5–10
  4: 10–20
  5: more than 20
11. After inhalation of hippy crack, how much would you say it affected you?
  Scale 1–10
  1: not at all
  10: completely affected
12. How many times would you say you would take hippy crack before it has an effect on you?
  Scale 1–5
  1: one or two
  2: 3–5
  3: 5–10
  4: 10–20
  5: more than 20
13. What was the duration of the effect?
  Scale 1–5
  1: less than a minute
  2: 2–5 min
  3: 5–20 min
  4: 20–30 min
  5: up to an hour
  If you answered NO (haven’t taken hippy crack)
14. If you haven’t taken hippy crack, what is the likelihood for you to take hippy crack in the next 3 months?
  Scale 1–5
  1: very likely
  5: very unlikely
15. If you would want hippy crack, how easy would it be for you in your opinion to obtain hippy crack?
  Scale 1–5
  1: very easy
  5: very difficult
16. If you have never taken hippy crack before, in your opinion, would you be more comfortable taking hippy crack on your own or among friends?
  ◻ On my own
  ◻ Among friends
17. If you have never taken hippy crack before and it was your first time taking hippy crack, how many times, in your opinion, would you take hippy crack in one sitting?
  Scale 1–5
  1: one or two
  2: 3–5
  3: 5–10
  4: 10–20
  5: more than 20
  **All, answer questions**
18. In your opinion, how harmful would you say hippy crack is?
  Scale 1–10
  1: not harmful at all
  10: extremely harmful
19. Are you aware of any of the side effects of hippy crack? yes or no
20. In your opinion, how much would you say is too much hippy crack in one go?
  Scale 1–10
  1: one is enough
  10: should be no limit
21. Alex is at a house party with friends, it has reached 1 am and everyone has now settled down and is listening to music, when one of Alex’s friends brings out a whippet canisters (filled with nitrous oxide) and a small box of balloons. Everyone has already filled their balloons with nitrous oxide. One friend suggested that they should inhale hippy crack in the car, rolling up all the windows as it is a closed space and would be more fun as it will give have a greater euphoric effect.
  However, Alex remembers in a previous lecture that informed all of them how hippy crack could possibly effect the brain and possible risks upon inhalation, Alex also remembers that there is a greater risk if it was inhaled in a closed space.
  A balloon filled with nitrous oxide is handed to Alex and although some friends stay behind inhaling with the balloon Alex’s best friend heads out to the car inviting and pulling Alex along. Bearing in mind that this is Alex’s first time inhaling hippy crack, if you were Alex what would you do?
  ………………………………………………………………
  ………………………………………………………………
  ………………………………………………………………
  ………………………………………………………………
  ………………………………………………………………
  ………………………………………………………………
22. In your opinion, how important would you say it is to educate young people (16–25) about the effects of hippy crack?
  Scale 1–10
  1: not important at all
  10: extremely important
